# Biomechanical comparison of ultra‐high molecular weight polyethylene sutures of different thicknesses of the tensile strength for pullout repair of medial meniscal posterior root tear

**DOI:** 10.1002/jeo2.70055

**Published:** 2024-10-15

**Authors:** Hibiki Kakiage, Mikiko Handa, Tsuneari Takahashi, Katsushi Takeshita, Hirotaka Chikuda

**Affiliations:** ^1^ Department of Orthopaedic Surgery, Faculty of Medicine Gunma University Maebashi Japan; ^2^ Department of Orthopaedics, School of Medicine Jichi Medical University Shimotsuke Japan

**Keywords:** medial meniscus posterior root tears, porcine model, ultra‐high molecular weight polyethylene

## Abstract

**Purpose:**

Medial meniscus posterior root tears (MMPRT) are a risk factor for knee osteoarthritis. The predominant treatment for MMPRT is transtibial pullout repair, and loop suture remains the gold standard procedure. This study aimed to investigate the structural properties of the meniscus‐suture‐tibia (MST) complex after loop stitch using ultra‐high molecular weight polyethylene (UHMWPE) suture tape of different thicknesses.

**Methods:**

This study used 20 fresh porcine MMPRT model knees. All specimens were randomised into two treatment groups: (1) pullout repair using 1.3 mm suture tape (thin group, *n* = 10; 1.3 mm PERMATAPE, Mitek Sports Medicine) fixation and (2) pullout repair using 2.5 mm suture tape (thick group, *n* = 10; 2.5 mm PERMATAPE, Mitek Sports Medicine) fixation. The single‐loop stitch MS technique was utilised. The MST complex specimens were placed on a tensile tester. The structural properties of the MST complex (yield load, maximum load, liner stiffness, and elongation at failure) were identified.

**Results:**

No significant differences were found between the thin and thick groups in terms of maximum load (108.8 ± 49.6 vs. 90.1 ± 33.6 N; *p* = 0.34), yield load (43.8 ± 15.2 vs. 39.4 ± 15.5 N; *p* = 0.53), liner stiffness (12.6 ± 8.4 vs. 11.2 ± 5.5 N/mm; *p* = 0.45), and elongation at failure (27.1 ± 19.4 vs. 19.9 ± 10.0 mm; *p* = 0.32).

**Conclusion:**

The structural properties of the thickness of the different UHMWPE were comparable in MMPRT repair. Additionally, 1.3 mm PERMATAPE may demonstrate similar repair potential as 2.5 mm PERMATAPE.

**Level of Evidence:**

Level Ⅳ.

**Abbreviations**
MMPRT: medial meniscus posterior root tears
MS: meniscus‐suture
MST: meniscus‐suture‐tibia
UHMWPE: ultra‐high molecular weight polyethylene

## INTRODUCTION

The meniscus is a semilunar‐shaped tissue found within the knee joint. The posterior root of the meniscus secures the meniscus to the tibia and distributes axial loads to hoop stresses [3, 12, 16]. Medial meniscus posterior root tears (MMPRT) are a known risk factor for knee osteoarthritis because of the meniscus's hoop structure disruption, accounting for 10%–21% of all meniscus injuries [3, 10, 14, 16, 22]. MMPRT may appear after light activity, including descending stairs or walking [9]. Advanced age, female gender, sedentary lifestyle, obesity, varus knee alignment, and increased medial tibial slope are risk factors for MMPRT [3, 9, 10]. Treatment involves conservative therapy, such as administering hyaluronic acid injections, and surgery, including meniscectomy or repair surgery, such as the MMPRT pullout repair [3]. In recent years, MMPRT pullout repair has been performed to restore the native anatomy of the meniscus attachment [3].

However, early postoperative re‐displacement of the torn meniscus that was repaired may cause incomplete healing or a non‐anatomical position healing that alters the biomechanics of joint contact as observed in a non‐anatomical root repair [15]. The meniscus‐suture (MS) complex is one of the main factors contributing to postoperative displacements [5]. In that sense, loop stitches are preferred in the clinical setting due to their ease and superior stiffness and are considered the current standard [2, 6]. On the other hand, although simple stitches, modified Mason‐Allen suture patterns, and other complex configurations may show superior biomechanical properties, they can be challenging to perform arthroscopically [6].

To date, the structural properties of the MS complex using a 1.3 mm suture tape rather than a 2.5 mm suture tape remain unknown. We hypothesised that the MS complex after loop stitch using the 1.3 mm ultra‐high molecular weight polyethylene (UHMWPE) suture tape would demonstrate structural properties comparable to those using the 2.5 mm UHMWPE suture tape. Thus, the present study aimed to confirm this hypothesis.

## MATERIALS AND METHODS

### Study design

Animal experiments were performed in our institution's biomechanics laboratory and conducted under the Animal Care and Use Committee regulations at our institution. Ethical approval by the committee was waived due to the ex vivo study design. This study used 20 fresh porcine knees (age: 6 months, weight range: 100–120 kg, Tokyo Shibaura Zouki). Each specimen was randomly categorised into (1) pullout repair using 1.3 mm UHMWPE suture tape (thin group, *n* = 10; 1.3 mm PERMATAPE, Mitek Sports Medcine) fixation and (2) pullout repair using 2.5 mm UHMWPE suture tape (thick group, *n* = 10; 2.5 mm PERMATAPE, Mitek Sports Medcine) fixation.

### Surgical procedure for MMPRT

A total of 20 freshly porcine tibias and medial meniscus without any obvious macroscopic degenerative changes were collected. Porcine tissue has previously had morphometric and mechanical similarities to young adult human tissue [1, 19] and is commonly used to evaluate meniscal repair techniques [4, 6, 7, 8, 18].

**Figure 1:**
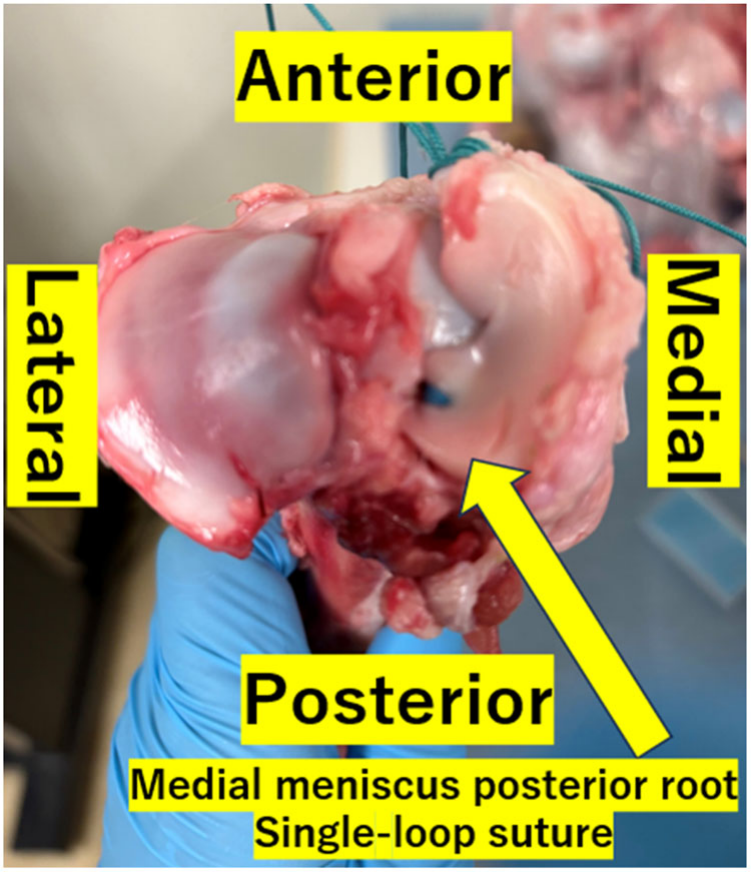
Postoperative specimen after pullout repair using 2.5 mm PERMATAPE.

**Figure 2:**
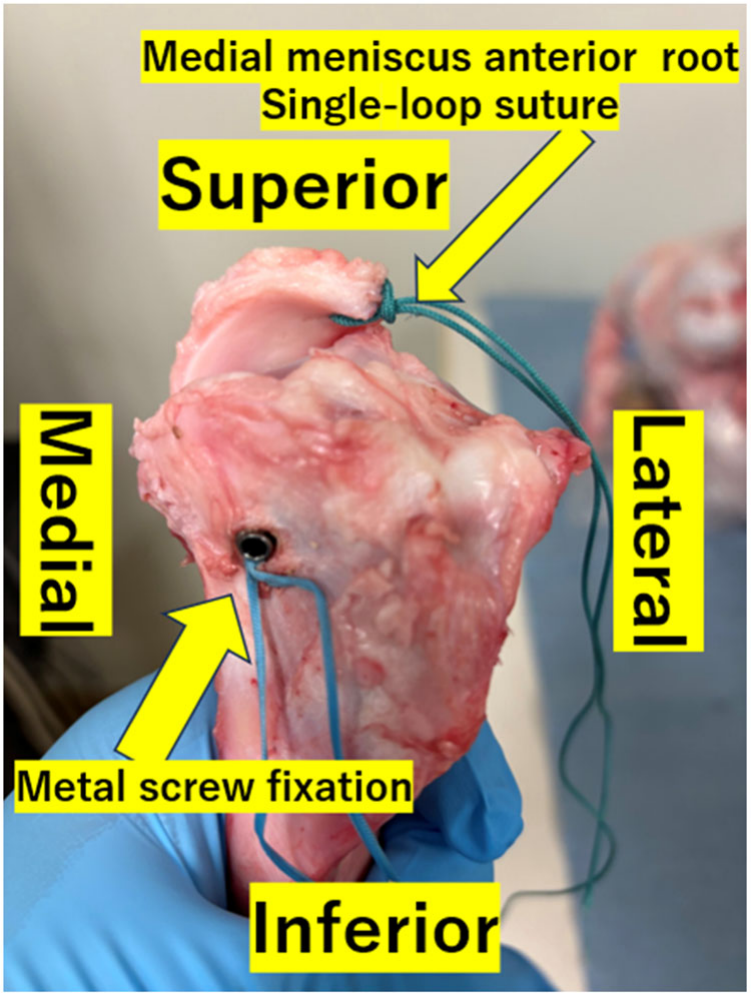
Postoperative specimen after pullout repair using 2.5 mm PERMATAPE.

The medial meniscus was released from the tibia by dissecting the meniscocapusular tissue and anterior meniscotibial ligament. The meniscus was completely detached from the tibial plateau by cutting the posterior meniscotibial ligament. A 4.5 mm drill was used to develop a transtibial tunnel from the posterior medial meniscus root footprint to the anteromedial cortex. A single‐loop suture using 1.3 mm PERMATAPE or 2.5 mm PERMATAPE was placed through the meniscus 5 mm medial from the resected edge of the posterior meniscus horn. We pulled the tape and passed it to the bone tunnel. A metal screw fixation with the manual max traction on the medial side of the tibia (Figures 1 and 2).

### Biomechanical testing of the meniscus‐suture‐tibia (MST) complex

**Figure 3:**
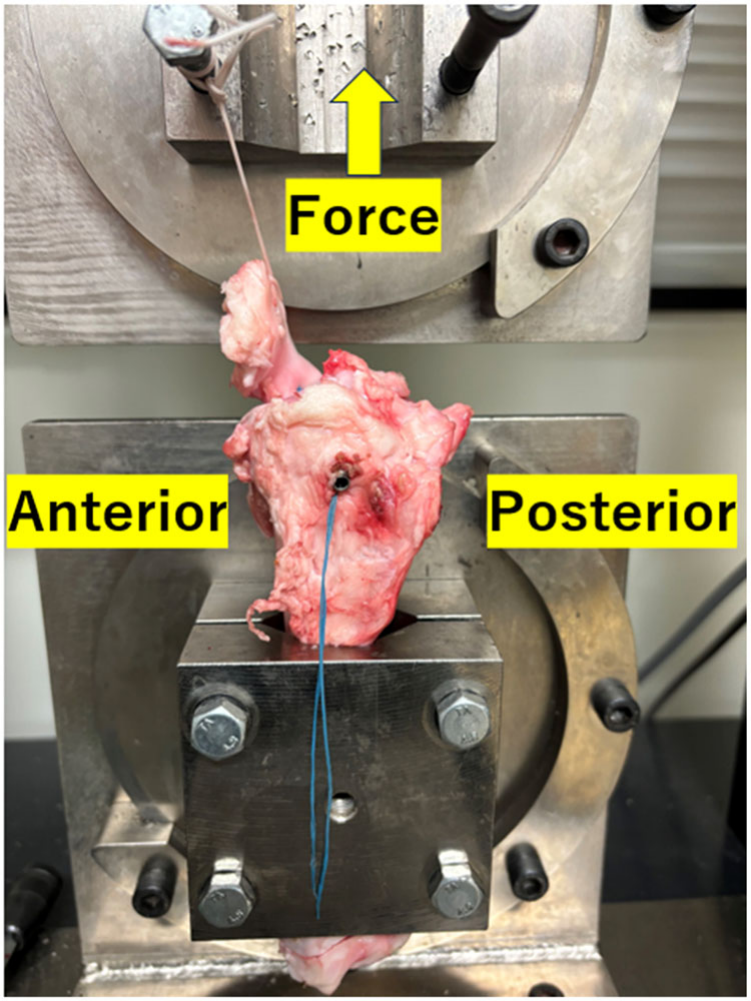
A prepared meniscus–tibia complex mounted on a tensile tester.

The prepared MST complex specimens were placed on a tensile tester (TENSILON RTG‐1250; Orientec Co., Ltd.) with a set of specially designed grips to apply a tensile load to the MST complex parallel to the long axis of the stitched suture materials (Figure 3). This measurement system was comparable to the one used in previous biomechanical studies utilising large animals [20, 21]. The specimen was preconditioned with a static preload of 5 N for 10 min before testing, followed by 10 cycles of loading and unloading (3% strain) with a crosshead speed of 20 mm/min. Afterward, each specimen was stretched to failure using the same conditions with preconditioning at a crosshead speed of 50 mm/min. These measurement conditions have been frequently utilised in previous studies with a large animal model [4, 20]. Failure modes were recorded. A load‐elongation curve was developed using specific software (TENSILON Advanced Controller for Testing, Orientec Co., Ltd.). The structural properties (yield load, maximum load, liner stiffness, and elongation at failure) of the MST complex were identified through software calculations. All tests were conducted at room temperature, and the MST complex was continuously kept moist using a saline solution.

### Statistical analysis

The Statistical Package for the Social Sciences software program (version 21.0; IBM) was used for all statistical analyses. All data from statistical analyses were presented as mean ± standard deviation. Student's *t*‐test was used to assess between‐group differences. A *p*‐value of <0.05 was considered significant.

## RESULTS

No significant differences were observed between the thin and thick groups in terms of maximum load (108.8 ± 49.6 vs. 90.1 ± 33.6 N; *p* = 0.34), yield load (43.8 ± 15.2 vs. 39.4 ± 15.5 *N*; *p* = 0.53), liner stiffness (12.6 ± 8.4 vs. 11.2 ± 5.5 N/mm; *p* = 0.45), and elongation at failure (27.1 ± 19.4 vs. 19.9 ± 10.0 mm; *p* = 0.32)(Table 1). Two specimens in the thin group were torn at the posterior root, whereas four specimens in the thick group demonstrated similar tearing at the posterior root, in terms of the failure mode observed in tensile testing. No significant differences were found between the two groups (Table 2).

**Table 1:** Outcomes of tensile testing.

Parameters	Thin group 1.3 mm PERMATAPE (*n* = 10)	Thick group 2.5 mm PERMATAPE (*n* = 10)	*p* value
Maximum load (*N*)	108.8 ± 49.6	90.1 ± 33.6	0.34
Yield load (*N*)	43.8 ± 15.2	39.4 ± 15.5	0.53
Linear stiffness (N/mm)	12.6 ± 8.4	11.2 ± 5.5	0.45
Elongation at failure (mm)	27.1 ± 19.4	19.9 ± 10.0	0.32

**Table 2:** Details of failures.

	Breakage of the anterior root	Breakage of the posterior root	Slipping of sutures	*p* value
Thin group 1.3 mm PERMATAPE (*n* = 10)	5	2	3	0.33
Thick group 2.5 mm PERMATAPE (*n* = 10)	4	4	2	

## DISCUSSION

The present study's main finding is the comparable biomechanical properties of repaired specimens MST complex using 1.3 mm UHMWPE or 2.5 mm UHMWPE. No differences were observed regardless of the UHMWPE tape thickness (1.3 mm and 2.5 mm). No difference in structural properties (maximum load, yield load, liner stiffness, and elongation at failure) was observed depending on the thickness of 1.3 mm and 2.5 mm UHMWPE tapes, the most important finding of this study. Improving the structural properties of the medial meniscus MST complex repair is critical. Takahashi et al. revealed that the ULTRATAPE (Smith&Nephew, MA) group demonstrated a significantly higher average maximum load than the 2‐0 UltraBraid (Smith&Nephew) group in MS complex porcine model [21]. Additionally, Kubo et al. revealed that the SUTURETAPE (Smith&Nephew) group exhibited a significantly higher maximum load than the 2‐0 UltraBraid group in the anterior cruciate ligament avulsion fracture fixation porcine model [13]. A greater contact area when the tape, rather than the thread, was utilised at the tissue insertion points may have contributed to improving the structural properties. However, the present study revealed no difference in structural properties regardless of the thickness of 1.3 mm and 2.5 mm UHMWPE tapes. The reason for the absence of significant differences in this study may be the comparison test between UHMWPE tapes rather than between UHMWPE tape and suture. The results of the current study reveal that 1.3 mm PERMATAPE may have the same repair potential as 2.5 mm PERMATAPE. Complex suture configurations could provide superior biomechanical properties but are technically challenging. Additionally, suturing the meniscus using thick tape may be technically difficult. This study shows that medial meniscus root repair with thin and thick tapes provides similar MST complex structural properties. The implants used in this study were priced as follows: 56,900 yen for 1.3 mm and 2.5 mm PERMATAPE.

### Limitations

The present study had several limitations. First, the porcine model was used, and, thus some of the results may not be directly transferrable to clinical practice in human patients. However, porcine knees are comparable to human knees in many ways, and many studies have used porcine knees [13, 17]. Second, a limited number of specimens and implants were available for use, which reduced the available sample size in each group. Third, the average age of the pigs during euthanasia was 6 months, with no obvious degeneration in all meniscus. MMPRT is prevalent in middle‐aged women, and aging and female sex are crucial factors associated with meniscal degeneration [11]. Therefore, these results cannot be directly applied to the clinical practice of the meniscus in middle‐aged humans. Fourth, the meniscus anterior root was pulled with a thread, which may have affected the rapture test. The results of this study provide valuable information about the efficacy of MMPRT repair using UHMWPE tape despite these limitations. Further specific studies are warranted to validate the conclusions of the present study.

## CONCLUSIONS

The structural properties of MMPRT repair using 1.3 mm and 2.5 mm UHMWPE suture tapes were comparable. Both sutures were deemed amenable for meniscus root repair.

## AUTHOR CONTRIBUTIONS

Tsuneari Takahashi and Mikiko Handa designed and conceived this study. Hibiki Kakiage and Mikiko Handa collected data. Hibiki Kakiage and Mikiko Handa analysed and interpreted the results and drafted the manuscript. Katsushi Takeshita and Hirotaka Chikuda substantially contributed to the revision of the manuscript drafts. All authors read and approved the final manuscript.

## CONFLICT OF INTEREST STATEMENT

The authors declare no conflicts of interest.

## ETHICS STATEMENT

Animal experiments were performed at our institution's biomechanical laboratory and conducted under the regulations of the Animal Care and Use Committee at Jichi Medical University. Ethical approval by the committee was waived due to the ex vivo study design. Issued protocol number: Not applicable.

## Data Availability

The authors have nothing to report.
